# Detection of colonisation by extended-spectrum beta-lactamase or carbapenemase producing *Enterobacterales* from frozen stool specimens

**DOI:** 10.1186/s13104-020-05279-9

**Published:** 2020-09-14

**Authors:** Pisey Tan, Shweta R. Singh, Bunsoth Mao, Konstantin Evdokimov, Vonthanak Saphonn, Li Yang Hsu, Paul Turner

**Affiliations:** 1 Cambodia Oxford Medical Research Unit, Angkor Hospital for Children, Siem Reap, Cambodia; 2 Saw Swee Hock School of Public Health, National University of Singapore, Singapore, Singapore; 3 University of Health Sciences, Phnom Penh, Cambodia; 4 Centre for Tropical Medicine and Global Health, Nuffield Department of Clinical Medicine, University of Oxford, Oxford, UK

**Keywords:** Frozen, Storage, Detection, Faeces, Antimicrobial resistance, *Escherichia coli*, *Klebsiella pneumoniae*

## Abstract

**Objective:**

To determine the impact of pre-culture ultra-low temperature (ULT, − 80 °C) storage of human stool specimens on recovery of Extended-Spectrum Beta-Lactamase (ESBL) or Carbapenemase (CPM) producing *Enterobacterales.*

**Results:**

Twenty stool specimens from a community-based household colonisation study in Cambodia were cultured fresh and after 4–5 days and ~ 6 months of ULT storage (as a slurry in tryptone soya broth–10% glycerol). Presumptive ESBL- and CPM-*Escherichia coli* isolates were detected in 19/20 (95%) and 1/20 (5%) freshly cultured specimens, respectively. The specimens yielded identical results when re-cultured after ULT storage at both time points. Detection of presumptive ESBL- and CPM-*Klebsiella / Enterobacter / Citrobacter* group was less frequent and slightly less stable over time. Comparison of antimicrobial susceptibility test profiles between pairs of *E. coli* and *K. pneumoniae* isolates from the two frozen culture time points revealed concordance in only 13/28 (46%) pairs, indicating likely colonisation by multiple strains. In conclusion, ULT storage of human stool specimens prior to culture appears to be an acceptable method for managing laboratory workflow in culture-based ESBL / CPM *Enterobacterales* colonisation studies in high prevalence settings.

## Introduction

The gastro-intestinal tract is a major reservoir for antimicrobial resistant bacteria in humans. High rates of gastro-intestinal tract colonisation by extended-spectrum beta-lactamase (ESBL) and carbapenemase (CPM) producing *Enterobacterales* have been reported in many studies [1].

Practical coordination of laboratory work for large colonisation studies can be a challenge, especially with community-based sampling where multiple specimens may be received by the laboratory at the end of the working day. Delays to processing specimens may result in sub-optimal results. However, storage at ultra-low temperatures (ULT) with delayed processing has been well validated for certain specimen types. For example, culture of fresh or − 80 °C stored nasopharyngeal swabs collected into skim milk–tryptone soya broth–glucose–glycerol (STGG) medium results in similar detection rates of *Streptococcus pneumoniae* [2]*.* Thus, pre-culture ULT storage of swabs in STGG has become an approved component of the WHO pneumococcal colonisation detection protocol [3].

There has been little published on the impacts of ULT storage of stool or rectal swabs specimens in the context of cultured-based colonisation detection. Declines in total anaerobes and *Bifidobacteria*, but no significant change in the *Enterobacteria* colony counts, were identified when comparing cultures of fresh and frozen stool specimens (stored in glycerol for 4 months) from 20 infants [4]. No differences were found in total aerobe colony counts in a comparison of seven adult stool specimens cultured fresh and after storage at -80 °C for 7 days [5]. However, there have been no evaluations specifically looking at the impact of ULT storage on detection of indicator antimicrobial resistant organisms, such as ESBL- and/or CPM-producing *Escherichia coli* or *Klebsiella pneumoniae.*

The aim of this study was to determine whether recovery of ESBL and/or CPM-producing *Enterobacterales* in faeces would be impacted by specimen storage at − 80 °C prior to culture.

## Main text

### Methods

As part of a community-based study to determine colonisation prevalence of multi-drug resistant bacteria, stool specimens were collected from members of 88 households living in Siem Reap, Cambodia. All culture work was done at the clinical microbiology laboratory Angkor Hospital for Children (AHC), Siem Reap. This laboratory participates in national and international (World Health Organization Invasive Bacterial–Vaccine Preventable Diseases) external quality assurance schemes [6].

For this evaluation, 20 sequential study stool specimens from five households were processed immediately on receipt in the laboratory. Faecal slurries were prepared by emulsifying a small amount stool, picked up using a sterile swab, in 1.5 ml of sterile tryptone soya broth (Oxoid, Basingstoke, UK)—10% glycerol storage medium. These slurries were cultured immediately and then following 4–5 days (Frozen #1) and 172–3 days (~ 6 months, Frozen #2) storage at − 80 °C.

For culture, 10 µL of fresh or defrosted faecal slurry was streaked onto both CHROMagar ESBL, for ESBL detection, and KPC, for CPM detection, plates (CHROMagar, Paris, France) and incubated overnight at 37 °C under aerobic conditions. The CHROMagar plates were prepared in-house following the manufacturer's instructions, including positive and negative quality control with appropriate American Type Culture Collection and in-house bacterial strains.

From the fresh specimen culture plates, presence or absence of pink (suspected *E. coli*) and blue (suspected *Klebsiella* sp., *Enterobacter* sp., *Citrobacter* sp.–KEC group) colonies was recorded. From the frozen specimen culture plates, colony presence or absence was recorded and then one pink and one blue colony (of the dominant morphotype, if colonial variation was noted) was picked from each plate for formal identification by matrix-assisted laser desorption/ionization time of flight mass spectrometry (MALDI-ToF; VITEK MS, bioMerieux, Marcy L’Etoile, France) and antimicrobial susceptibility testing (AST) by disk diffusion. The antibiotics tested were amoxicillin-clavulanate, ampicillin, cefpodoxime, ceftazidime, ceftriaxone, chloramphenicol, ciprofloxacin, gentamicin, meropenem, nitrofurantoin, and sulphamethoxazole-trimethoprim. The 2019 version of the Clinical Laboratory Standards Institute guidelines were used to interpret AST results [7].

Differences in detection of pink or blue colonies over time was assessed using the Chi-squared for trend test, with *p* values of < 0.05 being considered statistically significant. For comparison of AST profiles, "intermediate" results were re-assigned as "susceptible" to give a binary readout ("susceptible" or "resistant") for each isolate-drug combination.

## Results

Fresh culture of the stool specimens yielded pink colonies on 19/20 ESBL plates and 1/20 KPC plates. Blue colonies were detected on 10/20 ESBL plates and 1/20 KPC plates. Following freezing, the same pattern of pink colonies was identified on re-culture of the specimens at both time points (i.e. 100% concordance). In contrast, there was some variability in the detection of blue colonies between time points, although the differences did not reach statistical significance (Table 1). For blue colony detection on the ESBL plates, there were 15/20 specimens with agreement at all three time points. In the other five, loss of blue colonies following freezing occurred in one and blue colonies were detected only after freezing in the other four. One specimen had a blue colony on the fresh KPC plate, but this was not detected following freezing (Table 2). From frozen specimens culture plates, bacterial identity was confirmed as *E. coli* for all pink colonies. Of the blue colonies, 18/24 (75.0%) were *K. pneumoniae,* with the other six comprising *C. sedlakii* (n = 2), *E. hormaechei* (n = 2)*, E. kobei* (n = 1), and *Enterobacter* species (n = 1).

**Table 1:** Detection of presumptive extended spectrum beta-lactamase- (ESBL-) and carbapenemase- (CPM-) producing *Enterobacterales* from 20 stool specimens before and after storage at − 80 °C

Time point	Pink colonies*		Blue colonies^†^	
	ESBL plate	KPC plate	ESBL plate	KPC plate
Fresh	19 (95)	1 (5)	10 (50)	1 (5)
Frozen #1	19 (95)	1 (5)	12 (60)	0 (0)
Frozen #2	19 (95)	1 (5)	12 (60)	0 (0)
*p* value^‡^	1.0	1.0	0.5	0.2

*Indicative of *Escherichia coli*

^†^Indicative of *Klebsiella* sp., *Enterobacter* sp., or *Citrobacter* sp. (KEC group)

^‡^Chi-squared test for trend (column values)

**Table 2:** Individual stool specimen culture summary at each time point

Specimen	ESBL plate						KPC plate					
	Pink colonies			Blue colonies			Pink colonies			Blue colonies		
	Fresh	Frozen #1	Frozen #2	Fresh	Frozen #1	Frozen #2	Fresh	Frozen #1	Frozen #2	Fresh	Frozen #1	Frozen #2
1	Yes	Yes	Yes	Yes	No	No	No	No	No	No	No	No
2	Yes	Yes	Yes	No	No	No	No	No	No	No	No	No
3	Yes	Yes	Yes	No	No	Yes	No	No	No	No	No	No
4	Yes	Yes	Yes	No	No	No	No	No	No	No	No	No
5	Yes	Yes	Yes	Yes	Yes	Yes	No	No	No	No	No	No
6	No	No	No	No	No	No	No	No	No	No	No	No
7	Yes	Yes	Yes	Yes	Yes	Yes	No	No	No	Yes	No	No
8	Yes	Yes	Yes	No	No	No	No	No	No	No	No	No
9	Yes	Yes	Yes	Yes	Yes	Yes	Yes	Yes	Yes	No	No	No
10	Yes	Yes	Yes	Yes	Yes	Yes	No	No	No	No	No	No
11	Yes	Yes	Yes	No	Yes	Yes	No	No	No	No	No	No
12	Yes	Yes	Yes	No	No	No	No	No	No	No	No	No
13	Yes	Yes	Yes	No	Yes	Yes	No	No	No	No	No	No
14	Yes	Yes	Yes	No	Yes	No	No	No	No	No	No	No
15	Yes	Yes	Yes	Yes	Yes	Yes	No	No	No	No	No	No
16	Yes	Yes	Yes	No	No	No	No	No	No	No	No	No
17	Yes	Yes	Yes	Yes	Yes	Yes	No	No	No	No	No	No
18	Yes	Yes	Yes	Yes	Yes	Yes	No	No	No	No	No	No
19	Yes	Yes	Yes	Yes	Yes	Yes	No	No	No	No	No	No
20	Yes	Yes	Yes	Yes	Yes	Yes	No	No	No	No	No	No

"Yes" indicates detection of the colony type; "No" indicates the absence of the colony type

Antimicrobial susceptibility profiles were compared for 28 pairs of *E. coli* (n = 20) and *K. pneumoniae* (n = 8) isolates (i.e. the dominant species detected), cultured from the same specimen at the two post-ULT storage culture points (Table 3). In 13 pairs (46.4%), the AST profiles were identical indicating likely selection of the same strain on both cultures. In the other 15 pairs, between one and four AST results were different (median of two differences per pair). These differences were similar when comparing pairs of *E. coli* (same profile in 10/20, 50.0%) or *K. pneumoniae* (same profile in 3/8, 37.5%).

**Table 3:** Antimicrobial susceptibility testing summary by time point in paired *Escherichia coli* and *Klebsiella pneumoniae* isolates (i.e. isolates from the same specimen cultured from frozen at two discrete time points)

Pair ID	Organism	AST profile^†^–Frozen #1	AST profile^†^–Frozen #2	AST profile differences (n)
1	*Escherichia coli*	− − − − R − − − *	− − − − R − − − R	1
2	*Escherichia coli*	− – R R R * − − *	− − * R R R − − R	3
3	*Escherichia coli*	− R – R R − − − R	− R − * R − − − R	1
4	*Escherichia coli*	− − − − R − − − R	− − − − R − − − R	0
5	*Escherichia coli*	− * − * R R − − −	− R – R R R − − −	2
6	*Klebsiella pneumoniae*	− − − R R − − − R	− − − R R − − − R	0
7	*Escherichia coli*	− − − − R R − − R	− − − − R R − − R	0
8	*Klebsiella pneumoniae*	− − − R R − − − R	− − − * * − − − *	3
9	*Escherichia coli*	− R – R R * − − *	− * − * R R − − R	4
10	*Escherichia coli*	− − − − R R − − R	− − − − R R − − R	0
11	*Klebsiella pneumoniae*	− * − R R − * − R	− R – R R – R − R	2
12	*Escherichia coli*	R R – R R − − R R	R R – R R − − R R	0
13	*Escherichia coli*	− * − * R R − − R	− R – R R R − − R	2
14	*Klebsiella pneumoniae*	− R – R R – R − *	− * − R R − * − R	3
15	*Escherichia coli*	− R R R R R − − R	− R R R R R − − R	0
16	*Escherichia coli*	− − − R R − − − −	− − − * R − − − −	1
17	*Escherichia coli*	− – R R R R − − R	− – R R R R − − R	0
18	*Escherichia coli*	− − − R R − − − −	− − − R R − − − −	0
19	*Escherichia coli*	− * − R R R − − −	− R − * R * − − −	3
20	*Klebsiella pneumoniae*	− R – R R R * − R	− R – R R * R − R	2
21	*Escherichia coli*	− − − R R − − − R	− − − R R − − − R	0
22	*Escherichia coli*	− R R R R − − − *	− * R * R − − − R	3
23	*Escherichia coli*	− − − R R − − − R	− − − R R − − − R	0
24	*Klebsiella pneumoniae*	− − − R R − − − R	− − − * R − − − *	2
25	*Escherichia coli*	− − R R R − − − R	− − R * R − − − R	1
26	*Klebsiella pneumoniae*	− R − R R R − − R	− R − R R R − − R	0
27	*Escherichia coli*	− − − R R − − − R	− − − R R − − − R	0
28	*Klebsiella pneumoniae*	− R − − R − − − −	− R − − R − − − −	0

^†^The antimicrobial susceptibility (AST) profile consists of a " − " (susceptible / intermediate) or "R" (resistant) for each of the following antimicrobials (in order): amoxicillin-clavulanate, ceftazidime, chloramphenicol, ciprofloxacin, cefpodoxime, ceftriaxone, gentamicin, nitrofurantoin, meropenem, and sulphamethoxazole-trimethoprim. Mismatched susceptibility results within an isolate pair are labelled with asterisks "*". Ampicillin and cefpodoxime results were excluded as they were “R” throughout

## Discussion

This pragmatic study evaluated the impact of ULT storage of faecal slurries prior to culture to detect ESBL or CPM producing *Enterobacterales* using commercially available chromogenic media. No significant differences in crude presumptive detection rates for these organisms were found before and after ULT storage. At the individual level, the detection of suspected ESBL- or CPM-*E. coli* was unaffected by ULT storage. However, there was some variability in detection of suspected ESBL- or CPM-KEC group organisms over time. Also, analysis of pairs of *E. coli* and *K. pneumoniae* isolates cultured from the same frozen specimen at two different time points revealed variabilities in AST profiles. The most likely explanation for this is random selection of different colonising strains, since it is known that multiple strains of drug-resistant *E. coli* and *K. pneumoniae* may be carried concurrently [8]. A previous study from the AHC patient population identified up to 10 discrete *E. coli* strains, as defined by multi-locus sequence type, when 16 colonies were sequenced per rectal swab specimen [9]. Picking of multiple colonies per plate may have improved the chance of detection of identical strains at both time points although, similar to detection of multiple pneumococcal serotypes in nasopharyngeal swab specimens, this would dependent on the strain relative abundances [10].

In conclusion, ULT storage of faecal slurries for up to 6 months prior to culture appears to be an acceptable method for managing laboratory workflow in culture-based ESBL / CPM *E. coli*, and probably other *Enterobacterales,* colonisation studies in high prevalence settings.

## Limitations

The major limitation of the study is that colony densities were not recorded to determine whether yield of target organisms decreased over prolonged storage. Also, the prevalence of ESBL colonisation was found to be extremely high in the study population so results may not be transferable to studies conducted in populations with lower colonisation prevalence. Given the identification of target species with different AST profiles over time, further work is required to characterise the longitudinal stability of multiple colonising clones at − 80 °C. It may be that all clones are not equally stable during storage, either due to clone-specific factors or population density in the original specimen, leading to differences in recovery over time. Metagenomic sequencing based experiments would best determine the impact of ULT storage on relative abundance of specific clones over time and would help to determine if there is a "best before" date for culture of stored faecal specimens. Finally, given the slight variability in detection of KEC group organisms over time, a larger study may be warranted to confirm our findings for species other than *E. coli*.

## Data Availability

The datasets generated and/or analysed during the current study are available in the Figshare repository, https://figshare.com/articles/dataset/COMRU-NUS-UHS_ESBL-CPM-E_stool_data/12915842.

## Abbreviations

AHC: Angkor Hospital for Children
AST: Antimicrobial susceptibility test(ing)
CPM: Carbapenemase
ESBL: Extended spectrum beta-lactamase
KEC: *Klebsiella* Sp.—*Enterobacter* sp.—*Citrobacter* sp.
KPC: *Klebsiella pneumoniae* Carbapenemase
MALDI-ToF: Matrix-assisted laser desorption/ionization time of flight mass spectrometry
STGG: Skim milk—tryptone soya broth—glucose—glycerol
ULT: Ultra-low temperature
